# Supplementary material for: Macrophage internalization creates a multidrug-tolerant fungal persister reservoir and facilitates the emergence of drug resistance
Source: Nat Commun. 2023 Mar 2;14:1183. doi: 10.1038/s41467-023-36882-6 (PMC9981703; doi:10.1038/s41467-023-36882-6)
Supplement: Supplementary file 1 — Supplementary Information [file 41467_2023_36882_MOESM1_ESM.pdf]

Supplementary Figure 1. a. Fungal burdens were reduced by caspofungin treatment in the kidney but not in the spleen. **b.** Caspofungin concentrations were somewhat higher in the kidney than in the spleen, but greatly exceeded the MIC (0.25 μg/ml) in both organs. CSF: caspofungin. Data are presented as mean values +/- standard deviations. n = 5 independent biological replicates (i.e., mice) examined in one experiment.

Supplementary Figure 2. Starvation and oxidative stress rapidly induce micafungin tolerance in *C. glabrata in vitro*. MFG: micafungin. Data are presented as mean values +/- standard deviations. Two-sided t-test was used to calculate the p-value for the 5-minute timepoint. No adjustment for multiple comparisons was made because only one p-value was calculated. n = 8 independent biological replicates (cultures) examined over two independent experiments.

Supplementary Table 1. List of *C. glabrata* isolates used in the current study and their microbiological profiles.

Supplementary Table 2. Pilot study: Detection of clinical ECR *C. glabrata* colonies on YPD plates containing various micafungin concentrations.

Supplementary Table 3. CFU and ECR colony numbers obtained under intracellular and planktonic conditions.

Supplementary Table 4. CFU and ECR colony numbers obtained under intracellular and planktonic conditions in ROS detoxification mutants.

Supplementary Table 5. List of primers used in this study and their purpose.

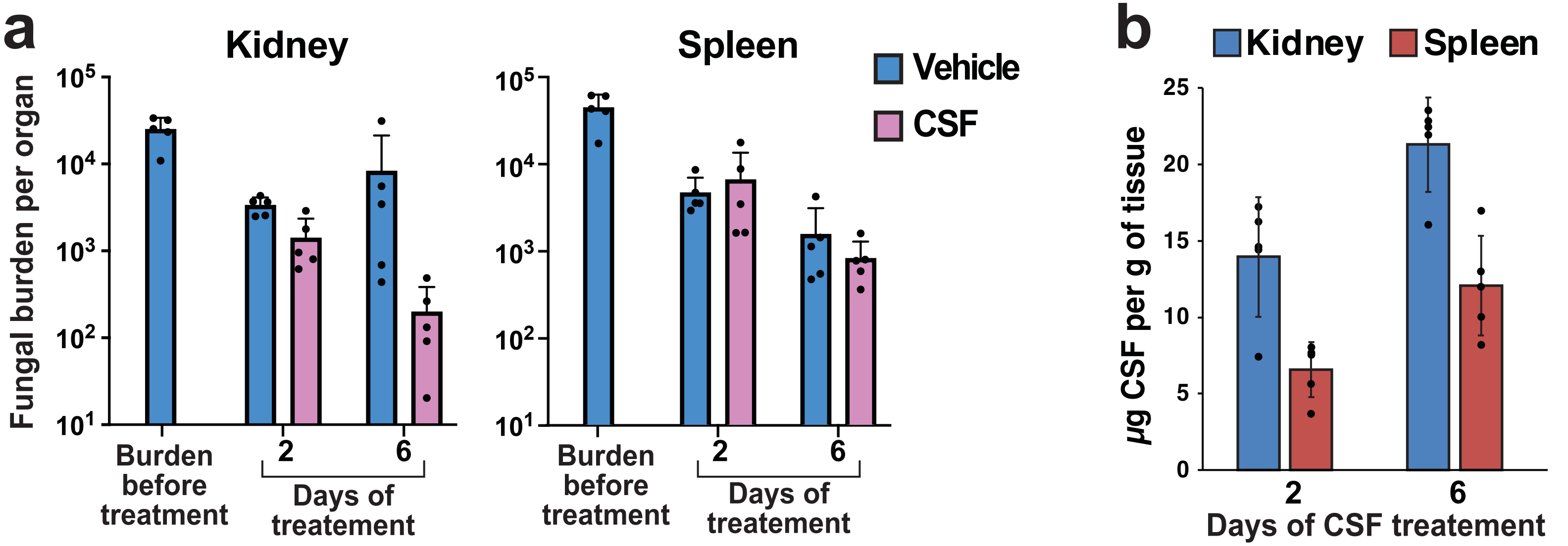
a
Kidney
Spleen
Vehicle
CSF
Fungal burden per organ
10^5
10^4
10^3
10^2
10^1
Burden before treatment
2
6
Days of treatement
b
Kidney
Spleen
μg CSF per g of tissue
25
20
15
10
5
0
Days of CSF treatement

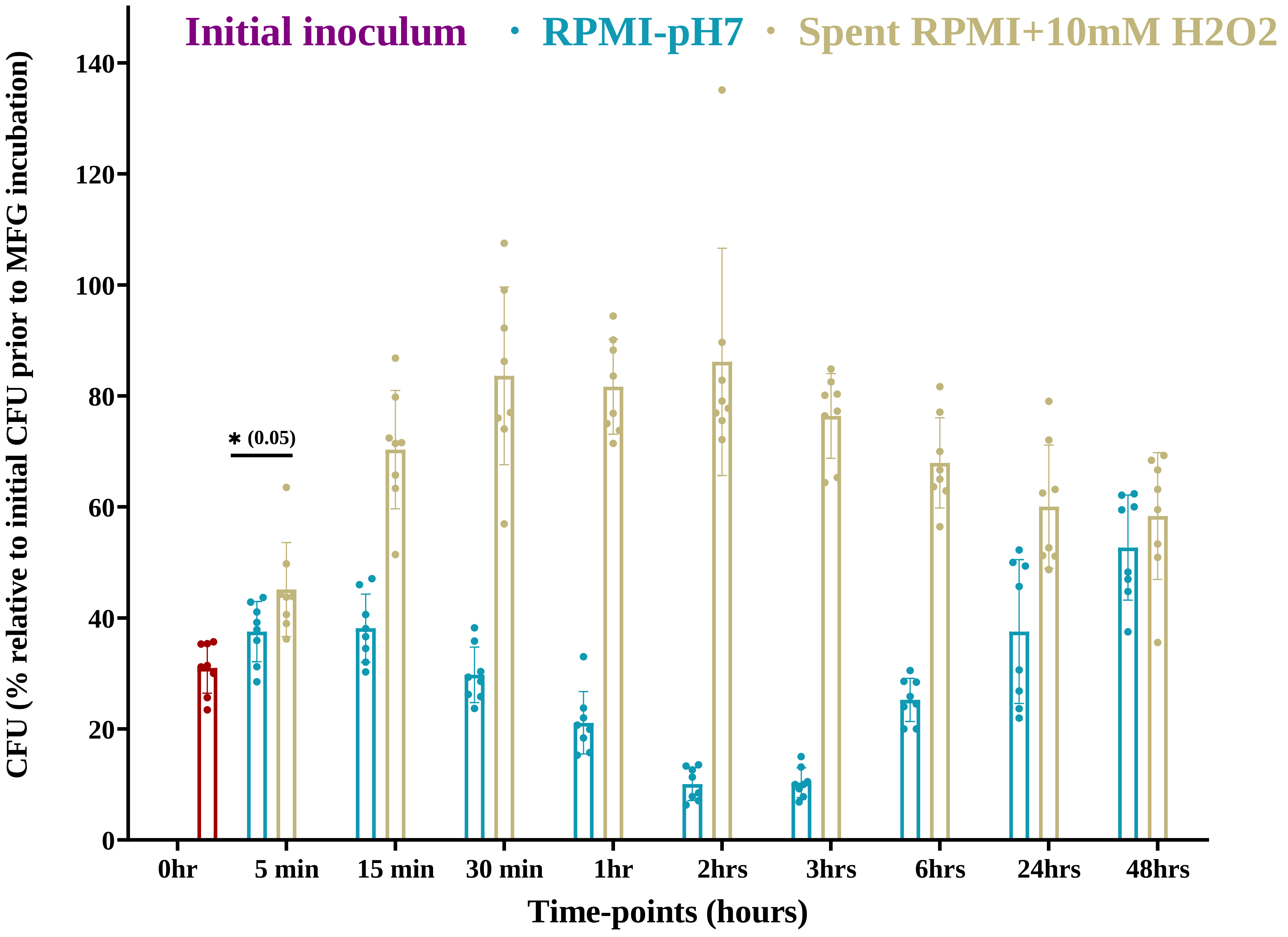

Initial inoculum
RPMI-pH7
Spent RPMI+10mM H2O2
CFU (% relative to initial CFU prior to MFG incubation)
0
20
40
60
80
100
120
140
* (0.05)
0hr
5 min
15 min
30 min
1hr
2hrs
3hrs
6hrs
24hrs
48hrs
Time-points (hours)

Supplementary Table 1. List of *C. glabrata* isolates used in the current study and their microbiological profiles

Strain #	Original identifier	Sequence type	Minimum inhibitory concentration (µg/ml)				Susceptibility profile
			Fluconazole	Micafungin	Anidulafungin	Amphotericin B	
5	Qatar 36	ST3	4	0.015	0.125	0.5	Susceptible
21	Qatar 38	ST15	4	0.015	0.25	1	Susceptible
24	ATCC 2001 (CBS 138)	ST15	4	0.015	0.125	0.5	Susceptible
25	CAS08-0205	ST10	4	0.015	0.125	1	Susceptible
35	DPL1021 (ATCC 90030)	ST10	4	0.015	0.125	1	Susceptible
36	CAS08-0092	STY	2	0.015	0.125	1	Susceptible
44	LB599-02	ST46	4	0.015	0.125	1	Susceptible

Supplementary Table 5. List of primers and their functions used in this study.

Oligo name	Sequence	Function
ICL1-F	5'-GAGCCTCTGATTCATATGACC-3'	Diagnosis of *ICL1* deletants
ICL1-R	5'-GTTACAGGGTAAGCGTAAGG-3'	
ICL1-UltF	5'GCTATTCACAGTTAGTCATTATTACAAACAAATCTTAACTACAAGAGCTATGTCTTTATCCTCAGCAGCTAGAGCTACCCCAGCCTTGAGGGCACAAGCTTGCTTGCCTCGTCCCCGC-3'	Amplification of NAT cassette, transformation
ICL1-UltR	5'AGAATATTAATAATTGTTAGAAAGATCCCATTTCATCCTATTTATTTCTTGCTTTCAATAGATTTTACCAATTCCTTGAACTTCTCGGTGGAGAAAGACTCTGGATGGCGGCGTTAGTATC-3'	
ICL1-gRNA	5'-TAATCAGCACCAGATTTCAA-3'	Transformation
CTA1-F	5'-CTTTGAACTGAACTATTGTTT-3'	Diagnosis of *CTA1* deletants
CTA1-R	5'-CAAAAAATCAACATTATAACGC-3'	
CTA1-Ult-F	5'-CTCTTATAAACTTTATCATTTACCTCATTGAATAGAGCTGTTTTACAGCACTTTACGTGGTTCAAAAGCGCACTTAGATCTTAGCAGTTGGGGCGGCTTGCCTCGTCCCCGCC-3'	Amplification of NAT cassette, transformation
CTA1-Ult-R	5'-AGATAACTTCCCACAATTGAAAAAAAACATGTCCGCTAATCCAACTAACACTTCCGATGTTAGAGGTGACAGAGTTGTCACTAACTCTACAGGTTACCCCTGGATGGCGGCGTTAGTATC-3'	
CTA1-gRNA	5'-CCAGGATGGGTAGTTACCCT-3'	Transformation
GRX2-F	5'-ATATAAGTAGCTTGGGAGTT-3'	Diagnosis of *GRX2* deletants
GRX2-R	5'-ATGTTTTCAATAAAGAATCTTATC-3'	
GRX2-Ult-F	5'-CAATTCTTTTATACTCTTGTCTAAACTCAAATGATCAGCATTGATTTTAGCAATCTAATTGTAATAATCGCCGTTTTGTTATTAACTCGTTATATATCCCGCTTGCCTCGTCCCCGCC-3'	Amplification of NAT cassette, transformation
GRX2-Ult-R	5'-TAGAAAAATATAAAAGTATTGTCAATTAGTTCTAGTTTAAGCATCAGCACGCTAAAATTAAGCCAAAACCTTTTGTAGCAATGGTTGTAGTTTACCGGCTGGATGGCGGCGTTAGTAT-3'	
GRX2-gRNA	5'-CAAGGCTGTCGTTCTAGAAT-3'	Transformation
MSN4-F	5'-GGATGCAACCAAAATGTA-3'	Diagnosis of *MSN4* deletants
MSN4-R	5'-GCCCTTGCATGATATATATTAG-3'	
MSN4-Ult-F	5'-GAAGCACTGATGATAGGTCAAGAAATCGTAAAAAAGCAACGCCAACTAGATAAAATAAGGAACACTGTCATTATTCTTTAGTTGTGGAAGTACTTCCCGACGCTTGCCTCGTCCCCGCC-3'	Amplification of NAT cassette, transformation
MSN4-Ult-R	5'-TAAGATAAAACAAAGGCCATACCAAACACAGATCAACAAGAACAGATGAGTGTGGAACAGGAGCCTAGCTCGTGGTCGACACAGATGGAAAACATGTTCTCTGGATGGCGGCGTTAGTAT-3'	
MSN4-gRNA	5'-CCAATTCCACGGTTTGAGTG-3'	Transformation
SKN7-F	5'- CTAGGACGATAAAACATATTGA-3'	Diagnosis of *SKN7* deletants
SKN7-R	5'- AGTAGCTCATAGCAGTATAC-3'	
SKN7-Ult-F	5'- CGGTCACGGTCAATACGTAATCTGATATTACCGGAATAGCAATTTATAAACGCTATAAAACTAATATGGACTACGAAGTTAATGCGAATCAAATTCCCGTTCTGGGCGCTTGCCTCGTCCCCGCC-3'	Amplification of NAT cassette, transformation
SKN7-Ult-R	5'- AGTTTTACAGGAATAAAATACTAGTTAGAGATGCAAAATTACGTATGTTGCTTCTTAAATGCAGGCTGTCCATCATTTAACGGAGTCATACCAAGCGGAGATCGCTGGATGGCGGCGTTAGTAT-3'	
SKN7-gRNA	5'- GGTGATTGTCCACTTGGTAC-3'	Transformation
SOD2-F	5'-CCAGGTTGTAGTCTATTTTAA-3'	Diagnosis of *SOD2* deletants
SOD2-R	5'- CTACCTTGCACAATACATAT-3'	
SOD2-Ult-F	5'- GTGCTTACTTTAGGTGCGAATATGTAGAGAGAGTTGAAGAAAAAATTAGCTAATCTATTTAGTGACTTTGGATGTTTCGTACCTCTTCGCAGCTTCCGCTTGCCTCGTCCCCGCC-3'	Amplification of NAT cassette, transformation
SOD2-Ult-R	5'- CACGGACGTTCACCGAAAGTACAACATCTAACTTGACTTTGATAGAAATAACAGAGGTATACATTACAAGGAAACGATGTTGTCTACGTCTAGGCTGGATGGCGGCGTTAGTAT-3'	
SOD2-gRNA	5'- GATCTGCTGGCCAAGAGCAC-3'	Transformation
YAP1-F	5'- GCGATAGGTTTTAGTATACATG-3'	Diagnosis of *YAP1* deletants
YAP1-R	5'- CCAATATGCAACAGTAACT-3'	
YAP1-Ult-F	5'- GAGATAGTCTGCCATGTCCATTTTTACATGATTTTAAGTATGCCAGTAACGAGCGTCTTGAATGTGAATTTTGATCTACCGAGATAATATAGACAGGTGAGCTTGCCTCGTCCCCGCC-3'	Amplification of NAT cassette, transformation
YAP1-Ult-R	5'- CAACTTGAAGGTTTCTTAGACACGCCAAGGACAAGGACAGAGACAAGAACTAGGAAGTAAAAGATGGCTGAGGTGGATAACGGTGGCGCACAGAAGAGCACTGGATGGCGGCGTTAGTAT-3'	
YAP1-gRNA	5'- TTACTGCTTGTGCCAGGTAG-3'	Transformation

Mutation type and frequency and echinocandin resistant (ECR) colonies derived from planktonic *C. glabrata* cells exposed to RPMI containing 0.125µg/ml)*

cta1Δ			*grx2Δ*			*msn4Δ*		
**Frequency**	**Mutation**	**Positive ECR colony**	**Frequency**	**Mutation**	**Positive ECR colony**	**Frequency**	**Mutation**	**Positive ECR colony**
0.02043	S663P	227	0.00441	S663P	49	0.00018	S663P	4
0.051429	S663P	8000	0.1125	59del+S66	2000	0	0	0
0.006429	S663P	10000	0.016364	S663P	2000	0.0036	S663P	10000
0	0	0	0	0	0	0	0	0
0	0	0	0	0	0	0	0	0
0	0	0	0	0	0	0	0	0
0.004833	S663P	29	0	0	0	0.000142	S663P	4
0.000947	S663P	2	0	0	0	0.003536	S663P	11
0	0	0	0	0	0	0	0	0

skn7Δ			*sod2Δ*			*yap1Δ*		
**Frequency**	**Mutation**	**Positive ECR colony**	**Frequency**	**Mutation**	**Positive ECR colony**	**Frequency**	**Mutation**	**Positive ECR colony**
0	0	0	0	0	0	Not tested		
0	0	0	0	0	0	Not tested		
4.5E-07	R665S	1	0	0	0	Not tested		
0	0	0	0	0	0	0	0	0
0	0	0	0	0	0	0	0	0
0	0	0	0.036	S663P	2000	0.005294	R665G	600
0.006	S663P	26	0	0	0	0	0	0
0	0	0	0	0	0	0	0	0
0	0	0	0	0	0	0.030508	R665G	1200

Mutation type and frequency and echinocandin resistant (ECR) colonies derived from intracellular *C. glabrata* cells exposed to RPMI containing 0.125µg/ml)

CBS138-WT			*cta1Δ*			*grx2Δ*		
**Frequency**	**Mutation**	**Positive ECR colony**	**Frequency**	**Mutation**	**Positive ECR colony**	**Frequency**	**Mutation**	**Positive ECR colony**
0	0	0	7.76E-06	S663P	10	9.09E-07	S663P	1
4.86E-06	R665G	1	0.000101	S663P	28	0.000003	S663P	1
0	0	0	0.001241	59del+S66	40	0	0	0
0	0	0	0	0	0	0	0	0
0	0	0	0	0	0	0	0	0
0	0	0	0.00001	S663P	2	0	0	0

msn4Δ			*skn7Δ*			*sod2Δ*		
**Frequency**	**Mutation**	**Positive ECR colony**	**Frequency**	**Mutation**	**Positive ECR colony**	**Frequency**	**Mutation**	**Positive ECR colony**
0	0	0	0	0	0	0	0	0
2.42E-05	7	S663P	0	0	0	0	0	0
0	0	0	8.41E-06	R665S	1	0.00012	20	F659del
0	0	0	0	0	0	0	0	0
0	0	0	0	0	0	0	0	0
0	0	0	0.000771	S663P	120	0	0	0

yap1Δ		
**Frequency**	**Mutation**	**Positive ECR colony**
0	0	0
0	0	0
0	0	0
0	0	0
4.81E-05	R665G	14
0	0	0

Mutation type of *C. glabrata* cells exposed to RPMI containing either micafungin (0.125µg/ml) or AMB (2µg/ml)

Micafungin (0.125µg/ml) for 24 hours, followed by micafungin (0.125µg/ml) for 48 hours

cta1Δ- MICA			*skn7Δ*- MICA			*yap1Δ*- MICA		
**Frequency**	**Mutation**	**Positive ECR colony**	**Frequency**	**Mutation**	**Positive ECR colony**	**Frequency**	**Mutation**	**Positive ECR colony**
0.051429	S663P	8000	0	0	0	0	0	0
0.006429	S663P	10000	0	0	0	0	0	0
0	0	0	4.5E-07	R665S	1	0.005294	R665G	600
0	0	0	0	0	0	0	0	0
0	0	0	0	0	0	0	0	0
0.000947	S663P	2	0	0	0	0.030508	R665G	1200

Micafungin (0.125µg/ml) for 24 hours, followed by AMB (2µg/ml) for 48 hours

cta1Δ- AMB			*skn7Δ*- AMB			*yap1Δ*- AMB		
**Frequency**	**Mutation**	**Positive ECR colony**	**Frequency**	**Mutation**	**Positive ECR colony**	**Frequency**	**Mutation**	**Positive ECR colony**
0	0	0	0	0	0	0	0	0
0	0	0	0	0	0	0	0	0
0	0	0	0	0	0	0	0	0
0	0	0	0	0	0	0	0	0
9.8E-05	S663P	1	0	0	0	0	0	0

* The WT strain yielded no ECR mutants under planktonic conditions

Supplementary Table 3. CFU and ECR colony numbers obtained under intracellular and planktonic conditions.

Macrophages

RepI

44			36			35			25			24-CBS138			21		
	CFU count	sitive colonies		CFU count	sitive colonies		CFU count	sitive colonies		CFU count	sitive colonies		CFU count	sitive colonies		CFU count	ositive colonies
24hrs	470000	0	24hrs	66500	0	24hrs	208000	0	24hrs	33500	0	24hrs	61000	0	24hrs	1505000	0
48hrs	336500	0	48hrs	20600	0	48hrs	87000	0	48hrs	12550	0	48hrs	16550	0	48hrs	144000	0
72hrs	264000	2	72hrs	17650	0	72hrs	53900	0	72hrs	12150	0	72hrs	16550	0	72hrs	66300	0
96hrs	83500	0	96hrs	10000	0	96hrs	16150	0	96hrs	3650	0	96hrs	5650	0	96hrs	35700	0
120hrs	84500	0	120hrs	8550	0	120hrs	15050	0	120hrs	2800	0	120hrs	6600	0	120hrs	31450	0

RepII

44			36			35			25			24-CBS138			21		
	CFU count	sitive colonies		CFU count	sitive colonies		CFU count	sitive colonies		CFU count	sitive colonies		CFU count	sitive colonies		CFU count	ositive colonies
24hrs	196000	0	24hrs	116000	0	24hrs	1105000	0	24hrs	71000	0	24hrs	85000	0	24hrs	245500	1 (R1378G)
48hrs	139000	0	48hrs	87500	0	48hrs	62000	0	48hrs	6000	0	48hrs	59000	0	48hrs	97500	3 (R1378G)
72hrs	90000	0	72hrs	42500	0	72hrs	42400	0	72hrs	4000	0	72hrs	40000	0	72hrs	55000	0
96hrs	77500	0	96hrs	10900	0	96hrs	15550	0	96hrs	6600	0	96hrs	8550	0	96hrs	36150	0
120hrs	54200	0	120hrs	7750	0	120hrs	13300	0	120hrs	2500	0	120hrs	6000	0	120hrs	34400	0

RepIII

44			36			35			25			24-CBS138			21		
	CFU count	sitive colonies		CFU count	sitive colonies		CFU count	sitive colonies		CFU count	sitive colonies		CFU count	sitive colonies		CFU count	ositive colonies
24hrs	840000	0	24hrs	222500	0	24hrs	260000	0	24hrs	33000	0	24hrs	180000	0	24hrs	370000	0
48hrs	167500	0	48hrs	29000	0	48hrs	38500	0	48hrs	6000	0	48hrs	18500	7 (S663F)	48hrs	71000	0
72hrs	78500	0	72hrs	7500	0	72hrs	23000	0	72hrs	6000	7 (R1378S)	72hrs	10500	0	72hrs	29500	0
96hrs	72500	0	96hrs	7800	0	96hrs	15000	0	96hrs	1650	0	96hrs	3450	0	96hrs	21500	0
120hrs	54000	0	120hrs	6450	0	120hrs	13500	65G)+12 (F6[illegible]	120hrs	1350	0	120hrs	6950	1 (W714L)	120hrs	20200	1 (S663F)

RPMI

RepI

44			36			35			25			24-CBS138			21		
	CFU count	sitive colonies		CFU count	sitive colonies		CFU count	sitive colonies		CFU count	sitive colonies		CFU count	sitive colonies		CFU count	ositive colonies
24hrs	110000	0	24hrs	4000	0	24hrs	10000	0	24hrs	10000	0	24hrs	10000	0	24hrs	2000	0
48hrs	100000	0	48hrs	8200	0	48hrs	580000	0	48hrs	5000	0	48hrs	7300	0	48hrs	88000	0
72hrs	161000	0	72hrs	20000	0	72hrs	180000	0	72hrs	10000	0	72hrs	63000	0	72hrs	1320000	0
96hrs	2420000	2 (R665G)	96hrs	42000	0	96hrs	80000	0	96hrs	324000	0	96hrs	50000	0	96hrs	2600000	0
120hrs	2100000	0	120hrs	50000	1 (S663F)	120hrs	120000	0	120hrs	300000	0	120hrs	81000	0	120hrs	2050000	0

RepII

44			36			35			25			24-CBS138			21		
	CFU count	sitive colonies		CFU count	sitive colonies		CFU count	sitive colonies		CFU count	sitive colonies		CFU count	sitive colonies		CFU count	ositive colonies
24hrs	64000	0	24hrs	14000	0	24hrs	4800	0	24hrs	25200	0	24hrs	6000	0	24hrs	13200	0
48hrs	203000	0	48hrs	25400	0	48hrs	271000	0	48hrs	50000	0	48hrs	12800	0	48hrs	35400	0
72hrs	100000	0	72hrs	12000	0	72hrs	95000	0	72hrs	36000	0	72hrs	90000	0	72hrs	140000	0
96hrs	1000000	0	96hrs	22000	0	96hrs	92000	0	96hrs	356000	0	96hrs	100000	0	96hrs	4000000	0
120hrs	1500000	0	120hrs	40000	0	120hrs	303000	0	120hrs	322000	0	120hrs	132000	0	120hrs	2500000	0

RepIII

44			36			35			25			24-CBS138			21		
	CFU count	sitive colonies		CFU count	sitive colonies		CFU count	sitive colonies		CFU count	sitive colonies		CFU count	sitive colonies		CFU count	ositive colonies
24hrs	144000	0	24hrs	15500	0	24hrs	8400	0	24hrs	14000	0	24hrs	23000	0	24hrs	17500	0
48hrs	178000	0	48hrs	13400	0	48hrs	11100	0	48hrs	48200	0	48hrs	18500	0	48hrs	174000	0
72hrs	190000	0	72hrs	53000	0	72hrs	54000	0	72hrs	10000	0	72hrs	65000	0	72hrs	490000	0
96hrs	980000	0	96hrs	32000	0	96hrs	100000	0	96hrs	220000	0	96hrs	120000	0	96hrs	4840000	0
120hrs	1700000	0	120hrs	50000	0	120hrs	115000	0	120hrs	280000	0	120hrs	140000	0	120hrs	3130000	0

Supplementary Table 2. Detection of clinical ECR *C. glabrata* colonies on YPD plates containing various micafungin concentrations

YPD plates containing 0.125µg/ml of micafungin

***FKS* mutant**	**HS1-Fks1**		**HS1-Fks2**				**Susceptible-Wildtype**	
**CFU/mutants**	**S629P**	**R631G**	**F659Y**	**S663P**	**F659V**	**S663F**	**WT- clinical**	**WT-CBS138**
**10**	5	2	4	7	6	5	0	0
**100**	60	30	70	82	78	63	0	0
**1,000**	720	533	678	834	759	724	0	0
**10,000**	TMTC	4100	TMTC	TMTC	TMTC	TMTC	0	0
**100,000**	TMTC	TMTC	TMTC	TMTC	TMTC	TMTC	0	0
**1,000,000**	TMTC	TMTC	TMTC	TMTC	TMTC	TMTC	0	0

YPD plates containing 0.5µg/ml of micafungin

***FKS* mutant**	**HS1-Fks1**		**HS1-Fks2**				**Susceptible-Wildtype**	
**CFU/mutants**	**S629P**	**R631G**	**F659Y**	**S663P**	**F659V**	**S663F**	**WT- clinical**	**WT-CBS138**
**10**	2	0	1	4	3	3	0	0
**100**	30	12	40	56	61	49	0	0
**1,000**	425	258	514	596	547	584	0	0
**10,000**	3154	1226	2856	4526	4158	3264	0	0
**100,000**	TMTC	TMTC	TMTC	TMTC	TMTC	TMTC	0	0
**1,000,000**	TMTC	TMTC	TMTC	TMTC	TMTC	TMTC	0	0

List of strains used for this experiment

**Strain #**	**Original identifier**	**Sequence type**	**Minimum inhibitory concentration (µg/ml)**				**Susceptibility profile**	**Fks amino acid substitution**
			**Fluconazole**	**Micafungin**	**Anidulafungin**	**Amphotericin B**		
2	CAS08-0725 (CMD00311)	ST3	64	0.5	2	1	Multidrug resistant	Fks2-F659Y
4	BG2	ST3	4	0.015	0.125	1	Susceptibile	WT
11	DPL155 (M234)	ST6	1	0.25	1	1	Echinocandin resistant	Fks2-F659V
27	CAS09-1437 (CGA01045)	ST10	64	2	1 or 0.5	1	Multidrug resistant	FKS1-R631G
34	DPL274 (3-CPH-W20800)	ST15	1	1	4	4	Echinocandin resistant	Fks2-F659S
38	DPL245 (1611)	STY	16	4	4	0.5	Echinocandin resistant	Fks1-S629P
41	DPL217	ST17	2	0.5	1	0.5	Echinocandin resistant	Fks2-S663F
24	ATCC 2001 (CBS 138)	ST15	4	0.015	0.125	0.5	Susceptibile	WT

TMTC = too many to count
